# Calcium-Rich Fly Ash as a Sustainable Supplementary Cementitious Material for Enhanced Sulfate Resistance and Durability of Cementitious Composites: Experimental and Microstructural Perspectives

**DOI:** 10.3390/ma18184238

**Published:** 2025-09-09

**Authors:** Nikolaos Chousidis, George Batis

**Affiliations:** Department of Materials Science and Engineering, School of Chemical Engineering, National Technical University of Athens, Iroon Polytechneiou 9, 157 72 Athens, Greece; batis@chemeng.ntua.gr

**Keywords:** fly ash, supplementary cementitious materials, sulfate resistance, reinforcement corrosion, sustainable repair materials

## Abstract

This study explores the potential of calcium-rich fly ash from the Ptolemais region in Greece as a partial cement replacement for improving sulfate resistance in cementitious composites. An integrated experimental program, combining mechanical testing, electrochemical corrosion monitoring and microstructural characterization, was designed to capture the progression of material properties over time and their impact on performance. The experimental results proved that, at early ages, incorporation of fly ash led to reductions in compressive, tensile and bond strengths, attributed to delayed pozzolanic reactivity. However, over prolonged curing, secondary reactions consumed portlandite and generated additional calcium silicate hydrate, refining the pore network and reducing permeability. These microstructural improvements were associated with enhanced mechanical performance, improved durability indices and markedly lower reinforcement corrosion rates. Bond tests further revealed a shift from brittle to a more ductile response, offering advantages for repair applications. These findings establish calcium-rich Ptolemais fly ash as a as a sustainable and promising supplementary cementitious material that substantially enhances the long-term durability and sulfate resistance of cementitious systems.

## 1. Introduction

The long-term durability of reinforced concrete elements embedded in or exposed to soil environments represents a complex and multifactorial challenge that demands a comprehensive investigation of the chemical and physical interactions occurring at the concrete-soil interface [1,2]. Unlike structures exposed solely to atmospheric conditions, subsurface or partially submerged infrastructure are subjected to a diverse suite of degradation mechanisms initiated by the surrounding soil matrix [3,4]. These mechanisms include external sulfate attack and carbonation, all of which are strongly influenced by the local environmental conditions such as moisture availability, temperature and oxygen diffusion.

Sulfate ions (SO_4_^2−^), originating from a combination of natural geological sources and anthropogenic activities—including industrial effluents, mining, agricultural runoff and the microbial degradation of organic matter—are consistently ranked among the most aggressive soil-borne agents affecting concrete durability [5]. The external sulfate attack mechanism initiates as sulfate ions infiltrate the concrete pore network, interacting primarily with calcium-bearing phases such as portlandite (Ca(OH)_2_) and monosulfate aluminate hydrates within the hydrated cement matrix [6,7,8,9]. These reactions yield expansive secondary products, notably gypsum and ettringite, which exhibit volumetric expansions up to an order of magnitude greater than the original phases [10,11].

Reinforcement corrosion in sulfate-bearing soils is predominantly an indirect consequence of chemical and physical degradation of the surrounding cementitious matrix [12,13]. By increasing porosity, inducing microcracking and decreasing alkalinity through chemical reactions, sulfate ions render the concrete less effective as a physical and electrochemical barrier, accelerating ingress of other aggressive species including chlorides and CO_2_ [14,15,16,17]. These factors accelerate reinforcement corrosion by destabilizing the passive film under aggressive exposure conditions [18,19,20].

The complexity of concrete behavior under sulfate exposure arises from the intricate interplay among chemical reactions, ion transport mechanisms, and microstructural evolution over time [21]. These investigations highlight decalcification and calcium leaching as key drivers of matrix weakening, leading to reduced cohesion, increased porosity and microcrack development. This compromised microstructure facilitates accelerated ingress, which in turn exacerbates corrosion of the embedded steel and accelerates mechanical degradation [22]. Given the extensive application of reinforced concrete in essential geotechnical and civil infrastructure, the implications of sulfate-induced degradation are profound. Addressing this knowledge gap is pivotal for enhancing infrastructure resilience and aligning with sustainability goals by prolonging service life and reducing maintenance costs.

Early identification of sulfate-induced deterioration is fundamental for the timely implementation of maintenance, repair, or replacement strategies, which can significantly reduce lifecycle costs and prevent catastrophic failures [23]. Consequently, a combination of macro- and microscopic diagnostic techniques is indispensable. High-resolution methods including SEM, XRD, differential thermal analysis (DTA) and spectroscopic approaches can detect subtle changes in porosity, crack initiation and phase evolution at early degradation stages, thus enabling proactive condition assessment [24,25].

Despite extensive research on sulfate attack, no standard accelerated test method currently exists to reliably assess concrete’s resistance under sulfate exposure, which limits comparability and practical evaluation. Additionally, interactions with other deterioration processes, such as partial saturation and wetting—drying cycles, remain insufficiently understood under realistic field conditions. Recent studies have proposed various accelerated approaches to address these challenges, including vacuum-assisted sulfate diffusion and pH-controlled environments, but consensus is lacking [26,27,28]. Moreover, systematic investigations focusing on early-stage manifestations of sulfate distress on mechanical and microstructural properties of cement pastes and mortars remain scarce [29]. These gaps limit the development of reliable durability models and the optimization of cement formulations for aggressive environments. Addressing these deficiencies necessitates well-designed experimental protocols that replicate complex exposure scenarios and capture mechanistic insights from nanoscale to macroscopic levels.

To address existing gaps, this study experimentally investigates the effect of 5% sodium sulfate (by cement mass) exposure on fly ash (FA) cementitious mixtures, focusing on early degradation indicators. Mechanical properties, porosity and microstructural changes are systematically examined. Emphasis is placed on the role of supplementary cementitious materials (SCMs) like fly ash, known to enhance sulfate resistance through pore refinement, reduction in reactive calcium hydroxide and stabilization of hydration products. The findings aim to guide material design for improved durability and mitigation of reinforcement corrosion in sulfate-rich soils.

Fly ash, a pozzolanic SCM rich in reactive silica and alumina, improves sulfate resistance by consuming portlandite through pozzolanic reactions to form additional calcium silicate hydrate gel (C–S–H), thereby lowering free calcium hydroxide levels vulnerable to sulfate attack [30,31,32,33]. This modification enhances matrix durability while contributing to sustainability by valorizing industrial byproducts and reducing Ordinary Portland cement (OPC) consumption and associated CO_2_ emissions [34]. Recent advances further highlight the potential of industrial byproducts as SCMs. Studies on the modulation of initial CaO/Al_2_O_3_ and SiO_2_/Al_2_O_3_ ratios in slag/fly ash-based geopolymers have demonstrated synergistic effects that improve stabilization mechanisms and mechanical performance of clay matrices [35,36]. Similarly, ternary blends of steel slag, fly ash and calcium carbide residue have been shown to effectively stabilize sludge for subgrade applications, enhancing both macro- and microstructural properties. These findings reinforce the relevance of exploring calcium-rich Ptolemais fly ash, emphasizing its dual role in improving durability and promoting sustainable construction practices.

Numerous studies [37,38,39] have demonstrated that fly ash incorporation promotes microstructural densification, lowers permeability and improves long-term durability under aggressive sulfate exposure, reinforcing its suitability as a supplementary cementitious material in demanding environments. Recent research has further explored alternative SCMs beyond fly ash. For instance, Liu et al. (2024) demonstrated improved thermal performance, frost resistance and pore structure of cement-based composites through binary modification with mPCMs/nano–SiO_2_ [40]. Similarly, Zheng et al. (2025) investigated the effect of carrier-encapsulated microbial calcium carbonate (CaCO_3_) on the performance of cement mortar [41]. These developments emphasize the ongoing search for novel additives to optimize composite behavior, reinforcing the relevance and novelty of our study on calcium-rich Ptolemais fly ash as a sustainable alternative.

The experimental results demonstrate that incorporating 5% Na_2_SO_4_ by mass in cement pastes induces early chemical attack symptoms, including surface scaling, disaggregation and fissure formation. These effects are notably more severe in mixtures without SCMs, such as Ordinary Portland cement, which have limited sulfate buffering capacity. Extensive microcracking from sulfate reaction products facilitates ingress of CO_2_, moisture and chlorides, increasing risks of electrochemical corrosion and carbonation-induced depassivation of reinforcement. These findings highlight the necessity of integrated material design and optimized mix formulations to improve the durability of concrete in sulfate-contaminated soils.

## 2. Experimental Set-Up

### 2.1. Raw Materials, Mix Proportions and Specimen Preparation

The raw materials employed in this study comprised Ordinary Portland cement (CEM I 42.5 N, Titan Cement, Kamari, Greece), potable tap water, limestone aggregates sourced from a local quarry in Greece and fly ash obtained from the lignite-fired power plant of Agios Dimitrios, Ptolemais, Greece.

In the present study, relatively low replacement levels of 5% wt. and 10% wt. by cement mass. Ptolemais fly ash was deliberately selected, targeting improvements in workability, reduction in hydration heat and enhancement of long-term pozzolanic reactivity. This decision reflects the intrinsic high-calcium composition of the ash, distinguishing it from conventional high-calcium (Class C) fly ashes, for which higher replacement levels (typically 20–40%) are commonly employed. Preliminary investigations revealed that higher substitution rates markedly reduce the portlandite content and disrupt the hydration equilibrium, potentially impairing early-age mechanical performance and increasing vulnerability to carbonation. Furthermore, these lower substitution levels are characteristic of repair mortars and non-structural cementitious composites, where the objective is to enhance durability while minimizing adverse effects on mechanical properties.

Chemical analysis (Table 1) confirmed the calcium-rich nature of the fly ash, with elevated CaO content and substantial silica and alumina fractions that drive pozzolanic reactions with calcium hydroxide during cement hydration. X-ray diffraction and thermal analysis indicated a significant amorphous phase, supporting high pozzolanic potential for microstructure refinement and durability. Minor oxides such as Fe_2_O_3_, MgO, K_2_O and Na_2_O likely enhance chemical stability, while moderate SO_3_ content requires consideration for durability due to sulfate resistance implications. Low Loss on Ignition and minimal insoluble residue reflect the fly ash’s high quality and suitability as an SCM.

Mixing water conformed to EN 1008 [42] standards, possessing neutral pH (7.2 at 25 °C) and negligible concentrations of chloride and sulfate ions, thereby ensuring no unintended chemical interactions during hydration or subsequent durability testing. A water-to-cementitious material ratio (w/cement + FA), ranging from 0.65 to 0.72 was employed across all mixtures. This ratio was established through preliminary rheological tests, which demonstrated that lower w/c values compromised workability in fly ash-containing mixes, complicating casting and compaction procedures and potentially inducing heterogeneities detrimental to mechanical and durability performance.

Specimen mixing protocols were tailored to the specimen type: concrete mixtures were prepared in a standard rotary drum mixer to ensure sufficient mixing energy and homogeneity at a practical scale, while mortar samples were mixed using a standardized laboratory mortar mixer (100L, Bormann Pro BEM1800, Stuttgart, Germany). Solids were dry-blended for two minutes to ensure uniform distribution, followed by gradual addition of mixing water and three minutes of wet mixing to achieve homogeneity and optimum fluidity. Fresh concrete and mortar were cast into pre-lubricated steel or plastic molds to facilitate demolding and mitigate surface defects. Compaction was carried out using a vibrating table (version C278, MATEST S.p.A., Treviolo, Italy) operating at 3000 Hz for 15 s, a procedure validated in preliminary trials for its efficacy in minimizing entrapped air and ensuring adequate consolidation throughout the specimen volume. Table 2 summarizes the contents of raw materials used in the study for evaluating the concrete’s properties.

**Figure 1:**
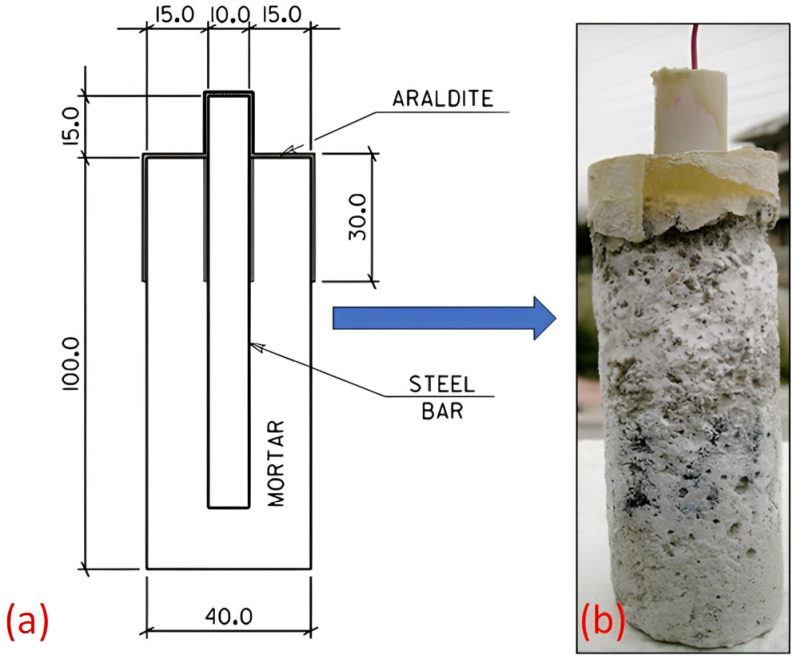
Schematic representation of the specimen configuration (**a**) and the mortar (**b**) exposed to Na_2_SO_4_. All dimensions are given in millimeters (mm).

To assess durability performance related to reinforcement corrosion and sulfate resistance, cylindrical reinforced mortar specimens were fabricated with dimensions of 50 mm diameter and 100 mm height, with an axially embedded Tempcore B500C steel rebar (SIDENOR, Almyros Magnisias, Greece) of 10 mm diameter and 100 mm length (Figure 1). A consistent cover thickness of 15 mm was maintained to replicate realistic concrete cover depths typically encountered in structural elements, ensuring relevance of diffusion and electrochemical phenomena. The mortar matrix comprised calcareous sand with particle size distribution between 250 μm and 4 mm and cement at a mass ratio of 3:1. The relatively high water-to-cement ratios (0.65–0.72) (Table 2) were intentionally selected to ensure adequate workability and fluidity characteristic of repair mortars and non-structural composites. Although these ratios exceed those typically employed in structural concretes (w/c ≈ 0.4–0.5), they result in lower early-age strength and higher porosity. This is because fly ash contributes predominantly through pozzolanic reactions at later ages.

The reinforcing bars were subjected to thorough cleaning and rust removal in accordance with ISO/DIS 8407 [43] specifications before embedding, involving immersion in hydrochloric acid (HCl) solution containing an organic corrosion inhibitor to remove native oxides and surface contaminants, followed by sequential rinsing with tap water, acetone and distilled water. Bars were then dried and precisely weighed with an analytical balance (±0.1 mg accuracy) to establish initial mass for subsequent corrosion rate calculations. The steel reinforcement consisted predominantly of iron (~99.17%), with minor elements including carbon (0.22%), sulfur (0.05%), phosphorus (0.05%) and nitrogen (0.01%). The low carbon equivalent (C_eq_ = 0.05%) signifies excellent weldability, reduced susceptibility to hydrogen embrittlement or cracking and consistent mechanical integrity under aggressive environmental conditions.

**Figure 2:**
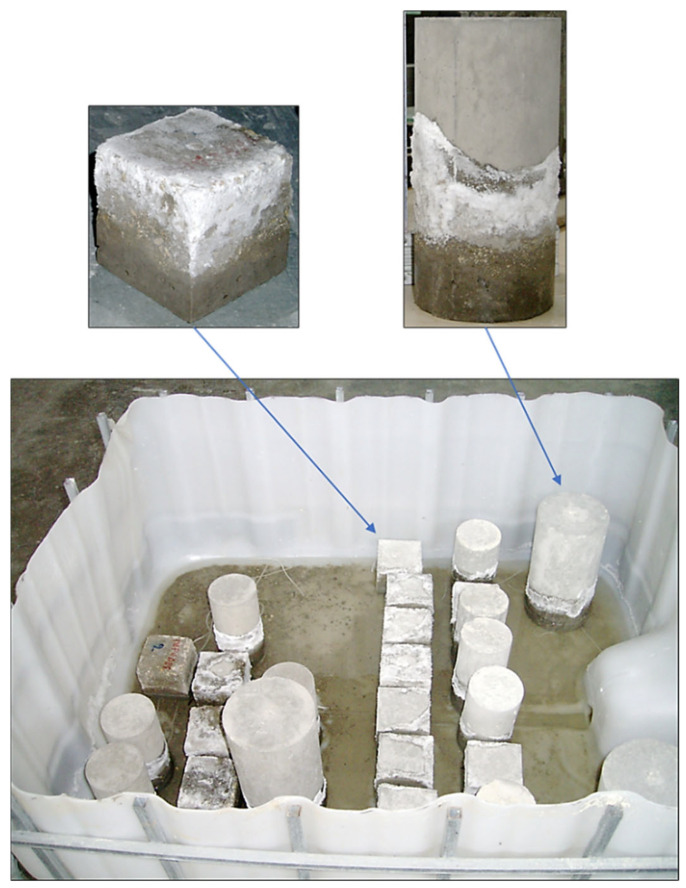
Cubic and prismatic concrete specimens partially immersed in 5% Na_2_SO_4_ solution.

For mechanical characterization, both cubic specimens (100 × 100 × 100 mm^3^) and cylindrical specimens (150 mm diameter × 300 mm height) were cast for standardized compressive and tensile strength tests according to EN 12390-3 [44] and EN 12390 [45] (Figure 2), respectively. Upon completion of casting, specimens were covered with impermeable sheets to mitigate moisture loss and maintained at ambient laboratory temperature for 24 h to allow initial set and early hydration. Subsequently, specimens were demolded and placed in a standard water curing tank at room temperature (25 ± 2 °C) with relative humidity exceeding 99%, ensuring continued hydration and minimizing microcracking due to autogenous and drying shrinkage. The curing period was set to 28 days, conforming to standard practice for mechanical and durability testing.

Durability specimens containing embedded steel were subjected to immersion in the 5% Na_2_SO_4_ solution under laboratory conditions replicating external sulfate attack scenarios. Sulfate ions penetrate the cementitious matrix primarily through diffusion and capillary suction, reacting chemically with calcium hydroxide (portlandite) and monosulfate aluminate hydrates to form expansive secondary phases such as ettringite and gypsum. The crystallization pressure of these phases induces microstructural damage through increased porosity, microcracking and eventual loss of mechanical integrity. This complex interaction between chemical attack and reinforcement corrosion highlights the necessity of evaluating these phenomena simultaneously under realistic exposure conditions.

### 2.2. Tests and Methods

#### 2.2.1. Mechanical Tests and Methods

**Figure 3:**
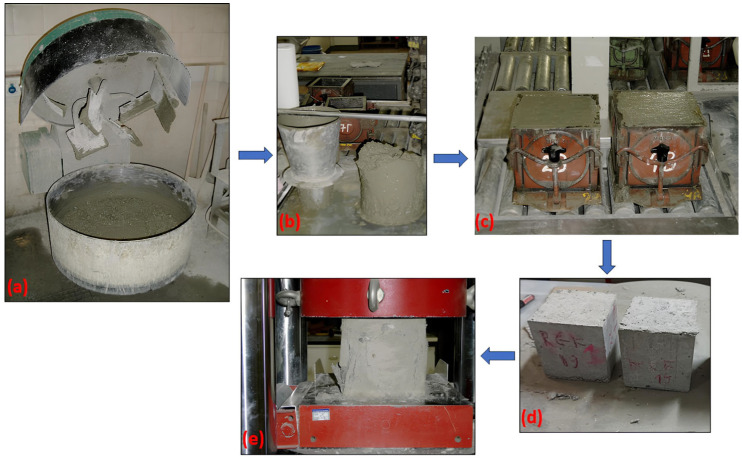
Experimental procedure for concrete testing: (**a**) mixing of raw materials, (**b**) slump test, (**c**) casting, (**d**) hardened specimens and (**e**) uniaxial compression test. Arrow denotes the sequence of steps.

The specimens were prepared following standardized mixing and curing procedures and with fly ash replacement levels of 0% (reference specimens), 5% and 10% by weight of cement. For each curing age, three specimens per mixture were tested to ensure reproducibility and statistical validity. All mechanical tests were conducted in a controlled laboratory environment at 25 ± 2 °C and relative humidity above 95%. The experimental procedure, illustrated in Figure 3, involved mixing, slump testing, casting, curing of hardened specimens and uniaxial compression testing, with the arrow indicating the sequence of steps.

The tensile performance of concrete was evaluated through the splitting tensile strength test (commonly referred to as the Brazilian test), which provides a reliable indirect measure of tensile capacity. Cylindrical specimens (150 mm in diameter and 300 mm in height) were prepared and tested, ensuring methodological consistency and reproducibility. During the test, a diametral compressive load was applied across the specimen’s curved surface, generating tensile stresses perpendicular to the load direction. Failure typically occurred along the loaded diameter due to splitting. This method is widely accepted for its simplicity and effectiveness in characterizing tensile strength—a critical property influencing crack resistance, structural integrity and the long-term durability of concrete. Tests were performed at multiple curing intervals to monitor the evolution of tensile strength over time, thereby enabling the assessment of hydration progression and potential susceptibility to environmental degradation.

Subsequently, compressive strength was assessed using the standard uniaxial compression test, on both reference and fly ash (FA)-modified mixtures. Testing was performed using a Technik Toni universal testing machine (Model 2031, Toni Technik, Berlin, Germany) with a maximum load capacity of 600 kN, sufficient to accommodate a broad range of strength values. A controlled loading rate of 0.5 MPa/s was employed to ensure consistent stress application and minimize rate-dependent variability in failure behavior. Compressive tests were performed at ages of 7, 28, 90, 120, 150 and 180 days, allowing for a comprehensive evaluation of the mechanical performance across early, intermediate and later hydration stages. This time-resolved testing protocol enabled the identification of strength development trends, the influence of SCMs and any deterioration phenomena arising from prolonged exposure to aggressive environments.

#### 2.2.2. Determination of Total Porosity via Mercury Intrusion Porosimetry (MIP)

The total porosity of cementitious mortar specimens was characterized using Mercury Intrusion Porosimetry (MIP), a well-established and widely employed method for quantitative analysis of pore structure in cement-based materials. This technique provides critical insight into pore size distribution, cumulative pore volume and connectivity within the pore network—parameters that strongly influence transport properties, durability and mechanical performance. Its applicability is particularly relevant for assessing modifications imparted by SCMs such as fly ash, which affect the pore refinement and connectivity through long-term pozzolanic reactions.

For each measurement, approximately 5 g of powdered mortar was obtained by carefully crushing and sieving selected fragments of representative specimens. The preparation protocol was designed to maintain sample homogeneity while minimizing the introduction of artificial densification or microstructural damage that could bias results. Prior to mercury intrusion, samples were oven-dried at 50 °C for 48 h and subsequently cooled within a desiccator to ambient temperature, thereby ensuring the removal of free and adsorbed moisture that could influence mercury penetration and pressure measurements.

Mercury intrusion tests were carried out using a Porosimeter 2000 (Carlo Erba, Milan, Italy), with applied pressures ranging from 1 to 2000 kg/cm^2^. The high-pressure porosimeter enabled intrusion pressures up to 455 MPa, allowing mercury to penetrate pores with diameters as small as approximately 3 nm, in accordance with the Washburn equation, which relates the applied pressure (P) to the pore throat radius (r):

(1) $$r=\frac{2\gamma cos\theta }{P}$$

where *γ* is the surface tension of mercury (485 mN/m), *θ* is the contact angle between mercury and the solid surface (taken as 140° for cementitious materials).

The experimental procedure involved incremental pressure application, recording the volume of intruded mercury as a function of pressure. From these data, both the differential and cumulative pore volume distributions were computed, allowing estimation of pore size distribution characteristics and total accessible porosity.

Total porosity (*φ*_total_) was defined as the ratio of the cumulative intruded mercury volume to the initial bulk volume of the sample, expressed as a percentage. This can be formalized as:

(2) $$\varphi_{total}\;\left(\%\right)=\left(\frac{V_{Hg}}{V_{sample}}\right)\times 10$$

where *V*_*p*_ is the cumulative volume of intruded mercury, *V*_*s*_ is the bulk volume of the sample, *V*_Hg_ is the cumulative volume of intruded mercury corresponding to accessible pores and *V*_sample_ is the bulk volume of the tested powdered material. It should be noted that MIP measures accessible porosity and may underestimate closed pores or those below resolution limits; nevertheless, it remains a robust method for relative comparisons among mixtures and curing ages.

The MIP results were analyzed to evaluate the effect of fly ash incorporation on pore refinement, cumulative porosity reduction, and the potential refinement of critical pore size ranges that govern ion transport. These microstructural changes were correlated with mechanical and durability performance metrics to elucidate the overall impact of SCMs on the behavior of cementitious matrices exposed to sulfate attack.

#### 2.2.3. Corrosion Evaluation of Steel Reinforcements

The corrosion rate of steel reinforcements embedded in mortar specimens was systematically monitored on a weekly basis over a total duration of 24 months. A total of 72 cylindrical specimens were prepared, equally divided into three groups: control specimens, specimens incorporating 5% FA and specimens with 10% FA replacement. Mortar mixtures were produced using calcareous sand, potable water sourced from the National Technical University of Athens (NTUA) supply network and CEM I 42.5 N. The water-to-cement ratio was maintained between 0.65 and 0.72 to ensure uniform matrix properties. Each cylindrical specimen measured 100 mm in height and 50 mm in diameter. Tempcore B500C steel reinforcement bars were embedded centrally at a depth of 20 mm from the specimen base to minimize premature corrosion initiation resulting from surface exposure.

The corrosion behavior of the embedded steel was evaluated using the Linear Polarization Resistance (LPR) technique, following the guidelines of ASTM G59-97 [46]. Three series of mortar specimens were partially immersed in a 5% Na_2_SO_4_ solution for 12 months to simulate aggressive sulfate exposure.

Electrochemical measurements were periodically conducted throughout the exposure period using a Potentiostat/Galvanostat (Model 263A, EG&G Princeton Applied Research, Oak Ridge, TN, USA). Data acquisition and processing were carried out using the instrument’s proprietary software. The electrochemical cell was configured with the reinforcing steel bar as the working electrode, the saturated calomel electrode (SCE) as the reference electrode and a carbon rod as the counter electrode. Polarization scans were performed within a ±15 mV potential range relative to the open-circuit potential (OCP) versus SCE, at a scan rate of 0.1 mV/s. The LPR method is based on the Stern–Geary equation, which relates the measured polarization resistance (*R_p_*) to the corrosion current density (*i*_corr_), providing a quantitative evaluation of the corrosion rate. This electrochemical approach allows for sensitive and non-destructive monitoring of corrosion progression in embedded steel, enabling the assessment of the protective effects of SCMs such as fly ash under sulfate-induced corrosion conditions.

The corrosion current density, *i*_corr_, is calculated from the polarization resistance (*R_p_*), using the Stern–Geary Equation:

(3) $$i_{corr}=\frac{B}{Rp}\;$$

where *B* is the Stern–Geary constant, which depends on the anodic and cathodic Tafel slopes (*b*_a_ and *b*_c_) is defined as:

(4) $$B=\frac{b_{a}b_{c}}{{2.303[b}_{a}+b_{c\;}]}\;$$

Typical values of the Stern–Geary constant (B) for steel in concrete range from 26 to 52 mV, depending on the electrochemical environment. The corrosion current density (*i*_corr_) is directly proportional to the corrosion rate and serves as a quantitative indicator of the severity of steel reinforcement corrosion within the cementitious matrix.

#### 2.2.4. Mass Loss Measurements of Steel

To quantify the corrosion-induced mass loss of steel reinforcement, cylindrical mortar specimens with embedded steel rebars were prepared. Prior to casting, the reinforcing steel bars (100 mm in length and 10 mm in diameter) were mechanically cleaned and derusted following a sequential washing protocol involving tap water, distilled water and acetone. Subsequently, the bars were dried and weighed using an analytical balance with a precision of ±0.1 mg, according to the guidelines of ISO/DIS 8407.3 [43], to determine the initial mass (*M*_initial_).

The cleaned rebars were then embedded axially within freshly mixed cement mortar specimens, which were cured under controlled laboratory conditions. Thereafter, the specimens were partially immersed in a 5% Na_2_SO_4_ solution to simulate a sulfate-induced corrosion environment.

At each retrieval, specimens were removed from the exposure solution and the embedded steel rebars were carefully extracted from the mortar matrix. Adherent corrosion products and residual cementitious material were removed using a standardized procedure involving rinsing with distilled water and acetone. The cleaned rebars were then dried to constant weight prior to reweighing to determine the final mass (*M*_final_).

The percentage mass loss, serving as a direct quantitative indicator of corrosion severity, was calculated using the following Equation:

(5) $$\Delta m\;(\%)=\frac{Minitial-Mfinal}{Minitial}\;\times 100\%$$

Equation (5) provides a direct and quantitative measure of the severity of corrosion experienced by the reinforcement steel under sulfate exposure. The method’s accuracy and reproducibility were ensured through adherence to strict cleaning and weighing protocols, enabling reliable assessment of corrosion kinetics and evaluation of the protective effect imparted by supplementary cementitious materials within the mortar matrix.

#### 2.2.5. Carbonation Depth Evaluation

Carbonation constitutes a critical durability concern in cementitious materials as it involves the gradual neutralization of the inherently alkaline pore solution by atmospheric carbon dioxide (CO_2_). This chemical process reduces the pore solution pH, typically from above 12.5 down to below 9.5, thereby compromising the stability of the passive oxide film that naturally protects embedded steel reinforcement. The consequent loss of passivation heightens the likelihood of corrosion initiation, posing significant risks to structural integrity and service life of reinforced concrete. Accordingly, assessing carbonation depth serves as an essential diagnostic tool for evaluating the extent of chemical degradation and as an early predictor of reinforcement depassivation risk.

In this study, the resistance of cylindrical mortar specimens to carbonation was evaluated using an accelerated carbonation test under controlled laboratory conditions. Following casting, all specimens underwent standard moist curing at 25 ± 2 °C and RH > 99% for 28 days to ensure complete and homogeneous hydration. Thereafter, specimens were partially immersed in a 5% Na_2_SO_4_ solution to simulate aggressive sulfate conditions encountered in the field.

At systematically selected exposure intervals (up to 30 months), specimens were retrieved from the sulfate solution and oven-dried at 105 °C to constant mass, ensuring removal of free moisture that could interfere with carbonation detection. Subsequently, each specimen was carefully split longitudinally to expose a freshly fractured internal surface representative of the mortar matrix cross-section.

To visually demarcate the carbonation front, a 1% phenolphthalein indicator solution in ethanol was uniformly sprayed onto the fractured surface. Phenolphthalein, a pH indicator, exhibits pink coloration in regions where pH exceeds ~9.5, marking uncarbonated alkaline zones. Conversely, carbonated areas, where the pH has dropped below this threshold due to CaCO_3_ formation via the reaction of atmospheric CO_2_ with calcium hydroxide (Ca(OH)_2_), remain colorless. This sharp color contrast facilitates precise measurement of carbonation penetration depth.

Quantitative carbonation depth measurements were performed at multiple locations along the exposed cross-section using a digital caliper with an accuracy of 0.1 mm. These measurements provide critical data on the progression rate and extent of carbonation under combined sulfate exposure, allowing correlation with microstructural changes, mechanical property evolution and corrosion risk assessment. Combining accelerated carbonation testing with sulfate immersion provides a robust framework to evaluate the interplay between carbonation-induced chemical changes and sulfate ingress, which is critical for predicting durability and service life of cementitious materials in aggressive environments. 

#### 2.2.6. Pull-Out Test

**Figure 4:**
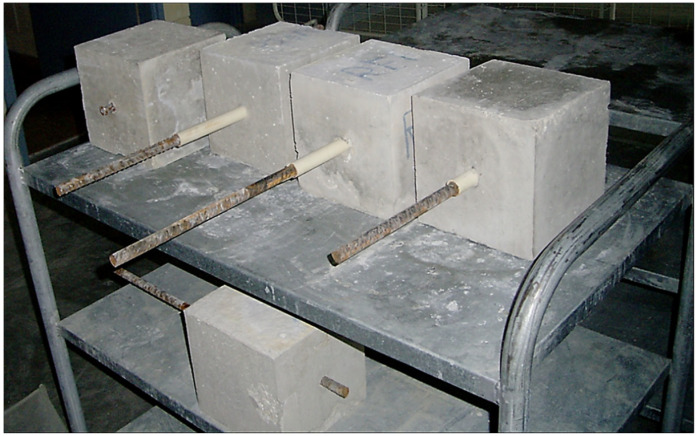
Cubic reinforced specimens (250 × 250 × 250 mm) for pull-out test.

The bond performance between concrete and steel reinforcement was assessed through pull-out testing at 28 days after casting (Figure 4). This test method provides critical insights into the interfacial behavior of steel within the surrounding cementitious matrix, which directly affects load transfer mechanisms in reinforced concrete structures.

In the experimental setup, 16 mm diameter reinforcing bars were centrally embedded into concrete cubes with an edge length of 250 mm. The embedded length of the bar was chosen to provide sufficient development length while avoiding premature splitting failure of the surrounding concrete. The rebar was axially aligned, with its protruding end gripped and subjected to uniaxial tensile loading at a controlled rate. The concrete specimen was firmly secured to prevent displacement or rotation during loading. In standardized bond testing, the mechanical response is predominantly dictated by the diameter of the reinforcing steel bars, whereas variations in the total embedded length have a comparatively insignificant effect on measured bond strength and overall test outcomes.

During testing, the applied tensile force and corresponding slip—defined as the relative longitudinal displacement between steel bar and concrete—were continuously recorded using a load cell and a displacement transducer (manufacturer details not available). Loading was continued until the maximum bond strength was achieved, thereby allowing the construction of a complete load–slip response curve. This curve characterizes the bond behavior, including initial stiffness, peak load and post-peak degradation, which are critical parameters for evaluating the effectiveness of reinforcement anchorage in concrete. To study the effect of sulfate exposure on bond behavior and durability, all specimens were first subjected to standard moist curing in a controlled curing chamber maintained at 25 ± 2 °C and >99% relative humidity for 28 days, to ensure adequate hydration. After curing, specimens were partially immersed in 5% Na_2_SO_4_ solution, following PN-EN 13295:2005, to simulate aggressive sulfate environments.

Three groups of mortar mixtures were investigated: a reference mix containing only OPC (CEM I 42.5N) as the binder and two modified mixes incorporating 5% and 10% Class C fly ash as partial cement replacements by weight. The inclusion of fly ash, sourced from the Ptolemais lignite-fired power plant, was aimed at enhancing the sulfate resistance and long-term durability performance of the mortar.

Performance was evaluated at 28 days through mass change measurements, visual inspection for cracking or deterioration and mechanical testing. The comparative results among the mixtures provide insight into the influence of fly ash content on sulfate-induced degradation and its effect on the bond strength between steel and concrete.

## 3. Experimental Results

### 3.1. Compressive Strength Development of Concrete

**Figure 5:**
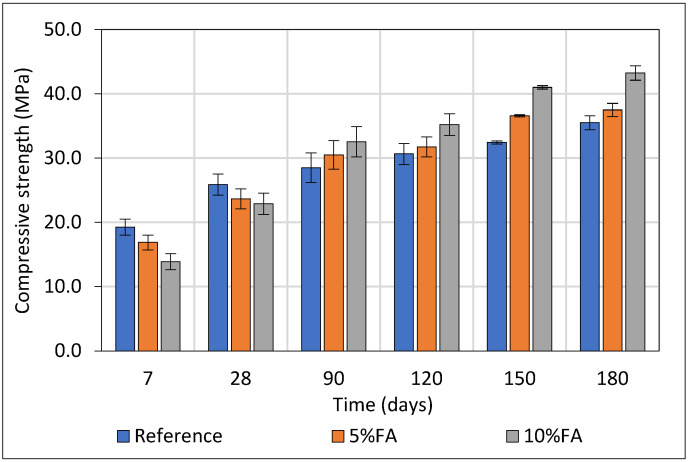
Axial compressive strength (MPa) values of concrete up to 180 days.

The evolution of compressive strength in mortar specimens incorporating Ptolemais FA at 5% and 10% cement replacement levels was systematically evaluated in comparison to a reference mixture without FA. Each group at every curing age was represented by three (3) tested specimens. As illustrated in Figure 5 at early age (7 days), all mixtures containing FA exhibited lower strength than the reference, with the 10FA and 5FA mixes achieving 13.9 MPa and 16.9 MPa respectively versus 19.2 MPa for the reference. This reduction (27.9% for 10% FA and 12.3% for 5% FA relative to the reference) can be attributed to the dilution effect associated with partial cement replacement and the limited early pozzolanic reactivity of the fly ash. At 28 days, this trend persisted but was less pronounced, with the 10FA and 5FA specimens reaching 22.9 MPa (−11.6%) and 23.7 MPa (−8.6%) respectively, compared to 25.9 MPa for the reference mix.

A significant strength gain was observed for FA-containing mixtures beyond 28 days, indicating the onset and progressive contribution of the pozzolanic reaction. At 90 days, the 10FA mix surpassed the reference by 14.2%, while the 5FA mix approached equivalence (−7.0%). At 180 days, the 10FA mixture reached 43.2 MPa, a 20.5% increase over the reference (35.5 MPa), confirming the continued strength gain, while the 5FA mix reached 37.5 MPa (+5.6%). Intra-group strength development also confirms the sustained contribution of FA: from 28 to 180 days, the 10FA mixture exhibited a strength increase of 89.1%, while the 5FA and reference increased by 58.5% and 37.3% respectively. This pronounced long-term enhancement in the 10FA mix is indicative of its superior pozzolanic activity, which progressively consumes calcium hydroxide and forms additional C–S–H, thereby densifying the microstructure.

These findings highlight the significant long-term benefits of incorporating fly ash at 10% replacement, primarily due to its progressive contribution to microstructural densification and mechanical enhancement. While early-age strength is reduced due to slower kinetics and cement dilution, the long-term performance surpasses that of conventional mixtures. These results underscore the suitability of Ptolemais fly ash for producing durable and sustainable cementitious systems where delayed strength gain is acceptable.

### 3.2. Splitting Strength Development of Concrete

**Figure 6:**
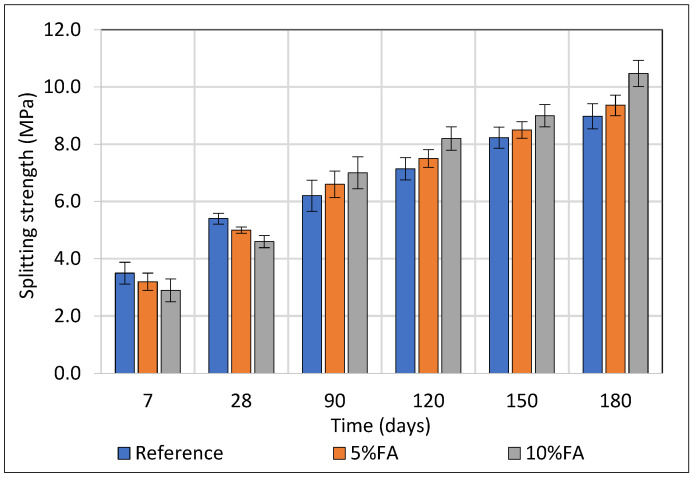
Flexural strength (MPa) values of concrete up to 180 days partially immersed in Na_2_SO_4_.

The splitting tensile strength of concrete specimens incorporating Ptolemais fly ash (FA) at 5% and 10% cement replacement levels was systematically evaluated against a reference mixture without FA over curing periods ranging from 7 to 180 days. For each mixture and testing age, a total of three specimens were examined. As presented in Figure 6, at early age (7 days), the 10FA and 5FA specimens exhibited reduced tensile strength values of 2.9 MPa and 3.2 MPa respectively, compared to 3.5 MPa for the reference mix, a reduction of approximately 17.1% and 8.6%. This early reduction can be attributed to the dilution effect caused by partial cement replacement and the limited early pozzolanic reactivity of the fly ash. By 28 days, the differences narrowed, with 10FA and 5FA achieving 85.2% and 92.6% of the reference tensile strength, respectively, indicating the onset of pozzolanic contributions enhancing matrix cohesion.

From 28 days onward, the 10% FA mixture exhibited a pronounced increase in tensile strength, reaching 10.5 MPa at 180 days, which corresponds to a 16.5% improvement over the reference mix at the same age. Similarly, the 5% FA mixture demonstrated a notable strength gain over time, achieving 9.4 MPa at 180 days, representing a 4.2% increase compared to the reference. In contrast, the reference mixture’s tensile strength increased more modestly by 27.4% over the same period, reflecting the absence of ongoing pozzolanic activity.

### 3.3. Total Porosity of Cement Mortars (MIP Method)

**Figure 7:**
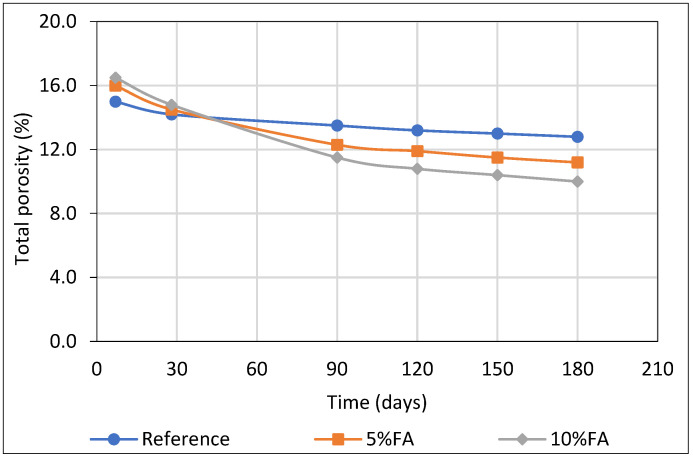
Total porosity (%) of cement mortars estimated by MIP method.

The total porosity of mortars incorporating 5% and 10% calcium-rich fly ash (FA) as partial cement replacement was evaluated over time and compared to a control (reference) mix without FA. Measurements were conducted using the Mercury Intrusion Porosimetry (MIP) method. At early age (7 days), the total porosity of the FA-containing mixes was slightly higher than that of the reference, with values of 16.0% and 16.5% for the 5% FA and 10% FA mixtures, compared to 15.0% for the reference (Figure 7). This initial increase can be attributed to the cement dilution effect, which delays early hydration. 

With progressing curing time, particularly after 90 days, the pozzolanic activity of FA became increasingly significant. The reactive silica and alumina in the fly ash reacted with portlandite (Ca(OH)_2_) to form additional C–S–H gel, effectively refining the pore structure and reducing pore connectivity. By 180 days, the 10% FA mixture exhibited the lowest porosity (10.0%), followed by the 5% FA mix and the reference, demonstrating the long-term microstructural benefits of incorporating calcium-rich fly ash.

In summary, porosity decreased more significantly over time in the mixes containing fly ash, highlighting the benefits of long-term microstructural densification. The results confirm that, despite a temporary increase in porosity at early ages, the incorporation of fly ash—particularly at a 10% replacement level—substantially contributes to pore structure refinement and enhances durability under sulfate-rich conditions.

### 3.4. Corrosion Current Density of Steel Reinforcement (LPR Method)

The corrosion behavior of embedded steel was further evaluated using the Linear Polarization Resistance (LPR) technique, which provides a rapid and non-destructive estimate of the corrosion rate. By applying a small potential perturbation and measuring the resulting current, LPR allows the calculation of the polarization resistance, which is inversely proportional to the corrosion rate. This method is particularly valuable for monitoring the temporal evolution of steel corrosion in mortar or concrete under different mix compositions and exposure conditions.

**Figure 8:**
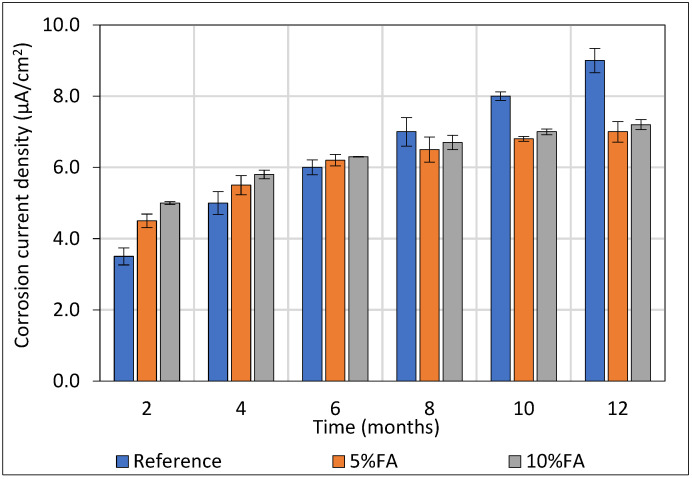
Corrosion of reinforcing steel (μA/cm^2^) embedded in cylindrical mortars and exposed to sulfate ions.

In the present study, the corrosion behavior of steel reinforcement embedded in mortar specimens was systematically investigated by measuring the corrosion current over time (Figure 8). The reinforcing bars had a diameter of 10 mm and a length of 100 mm, corresponding to a total exposed steel surface area of approximately 31.4 cm^2^.

Corrosion currents were normalized by the exposed steel surface area to obtain corrosion current density (icorr), enabling direct comparison between specimens and with literature data, thus facilitating the assessment of fly ash (FA) influence on corrosion behavior under sulfate exposure. The corrosion current density of steel reinforcement embedded in mortar specimens with 0%, 5%, and 10% FA replacement was monitored over a 12-month period in a 5% Na_2_SO_4_ solution.

At early ages (2–4 months), specimens containing FA exhibited slightly higher corrosion rates, attributed to slower hydration and delayed matrix densification in FA mixes. Specifically, at 2 months, icorr values were 2.0 μA/cm^2^ for the reference, 3.0 μA/cm^2^ for 5% FA, and 4.0 μA/cm^2^ for 10% FA specimens. This initial behavior is ascribed to the higher cement content in the reference mix, promoting faster early hydration and matrix densification, which temporarily mitigates sulfate ingress. From 6 months onward, a clear trend emerged: specimens incorporating FA demonstrated progressively lower corrosion rates compared to the reference. In particular, the 10% FA specimens showed a decrease in icorr from 1.5 μA/cm^2^ at 6 months to 0.8 μA/cm^2^ at 12 months, while the reference specimens exhibited a continuous increase in corrosion current density, reaching 7.0 μA/cm^2^ at 12 months. This long-term improvement is attributed to the pozzolanic reaction of fly ash with calcium hydroxide, forming additional C–S–H phase that refine the pore structure, reduce permeability, and limit sulfate ion ingress. Consequently, the embedded steel benefits from enhanced passivation and reduced corrosion activity.

The superior performance of the 10% FA specimens relative to the 5% FA mix reflects the higher availability of reactive silica, accelerating the pozzolanic effect and matrix densification. These observations corroborate previous studies reporting the beneficial role of FA in mitigating steel corrosion under aggressive environmental conditions over extended periods.

In summary, while FA addition may slightly compromise early-age corrosion resistance, its long-term effects are strongly advantageous, with 10% FA providing the most effective protection to steel reinforcement under sustained sulfate attack.

### 3.5. Corrosion Rate of Steel Reinforcement (Gravimetric Method)

**Figure 9:**
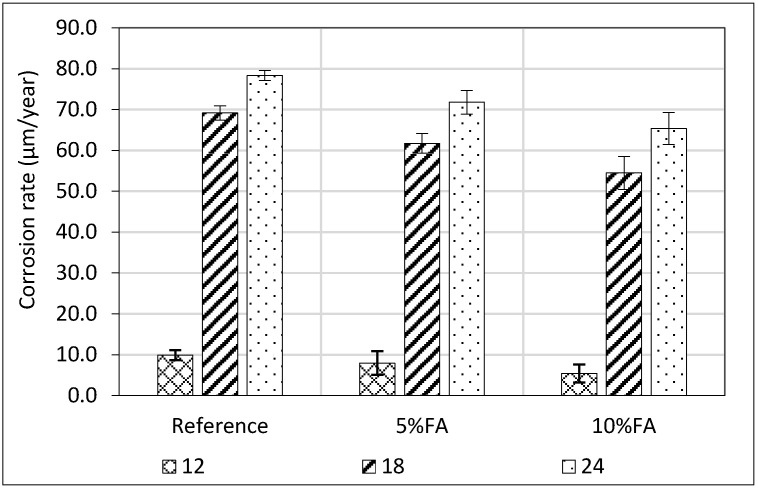
Corrosion rate (μm/year) of steel rebars embedded in mortars estimated by gravimetric method up to 24 months partially immersed to Na_2_SO_4_.

The analysis of mass loss (Δ*m*%) of embedded steel reinforcement reveals distinct differences among the mortar specimens containing 5% and 10% FA and the reference mixture without FA over exposure periods of 12, 18 and 24 months. For the mass loss measurements, three (3) specimens were tested for each group and curing age. Figure 9 illustrates the corrosion rate (CR) of steel rebars evaluated by gravimetric method.

At the 12-month mark, the reference mortar exhibited a corrosion rate of 9.90 μm/year. The addition of 5% and 10% FA resulted in substantially lower CR values of 7.98 μm/year and 5.41 μm/year, respectively. These reductions correspond to decreases of approximately 19.4% and 45.3% relative to the unmodified reference, underscoring the pronounced early-stage efficacy of FA in enhancing the microstructural integrity of the mortar. This performance improvement is primarily ascribed to the pozzolanic activity and filler effects of FA, which contribute to pore refinement and reduced connectivity, thus strengthening the microstructural barrier against corrosive agents.

Subsequent 18-month measurements indicated a marked increase in corrosion rates for all mixtures. The reference mortar’s CR surged to 69.17 μm/year, while the 5% and 10% FA mortars recorded values of 61.74 μm/year and 54.4750 μm/year, respectively. Although corrosion rates increased substantially at 18 months for all mixtures, FA-containing mortars continued to exhibit significantly lower corrosion rates than the reference, maintaining reductions of 10.7% and 21.2% for the 5% and 10% FA mixes, respectively. Corrosion rates further increased at 24 months across all specimens, yet FA mixes still showed significant corrosion reductions of 8.4% and 16.6% for 5% and 10% FA, respectively, compared to the reference.

The mitigation of corrosion rates in FA mortars is mainly due to pozzolanic reactions producing additional C–S–H gel, which densifies the pore structure and reduces permeability, limiting ingress of corrosive agents. The fine particle size of FA further acts as a microfiller improving matrix continuity. Although the consumption of Ca(OH)_2_ may slightly lower the pore solution pH, the overall microstructural refinement effectively maintains steel passivation.

In conclusion, Ptolemais fly ash at 10% replacement significantly improves long-term corrosion resistance of lime-based mortars, delaying corrosion initiation and mitigating its severity, highlighting its potential as a durable additive for restoration mortars.

### 3.6. Carbonation Depth of Cement Mortars

The carbonation depth of mortar specimens was evaluated using the phenolphthalein indicator method, providing a simple and effective way to assess CO_2_ penetration into the cementitious matrix. This test is widely employed to monitor its durability and evaluate the effectiveness of SCMs in mitigating carbonation-induced steel corrosion.

**Figure 10:**
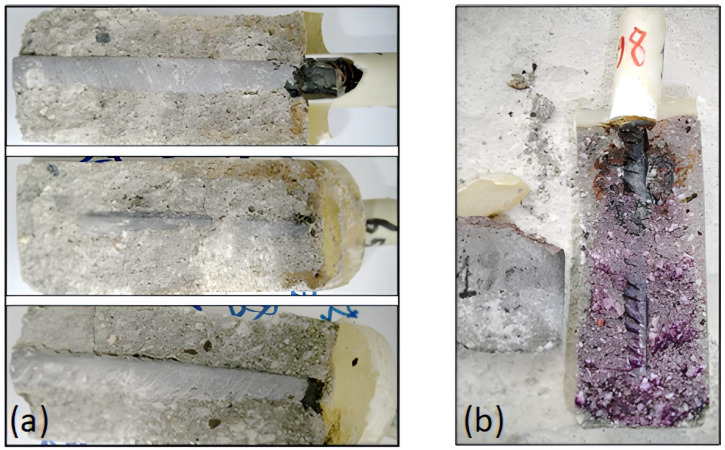
Cementitious mortar specimens after exposure to Na_2_SO_4_ (**a**) and corresponding phenolphthalein staining indicating carbonation depth (**b**). Purple indicates uncarbonated, colorless indicates carbonated areas).

The progression of carbonation depth over time revealed a clear dependence on the FA content in the mortar mixtures (Figure 10). After 30 months, the 10% FA specimens exhibited the smallest carbonation depth, followed by 5% FA, with the reference showed the highest penetration. This trend is substantiated by the calculated carbonation coefficients *k*_*c*_, derived from the linear relationship between carbonation depth and the square root of exposure time. Specifically, the estimated carbonation coefficients (*k*_c_) were 1.46, 2.27, and 3.21 mm/√month for the 10% FA, 5% FA, and reference specimens, respectively.

**Figure 11:**
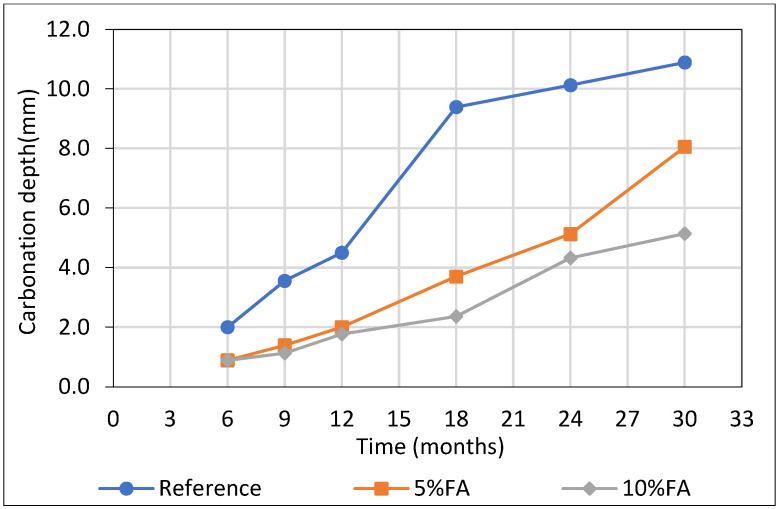
Carbonation depth estimation in cement mortars after sulfate attack.

Figure 11 shows the carbonation depth in reinforced cement mortars with and with-out FA after immersion in 5% Na_2_SO_4_. The reduction in carbonation rate observed in the 10% FA specimens represents a 54.5% decrease in k_c_ relative to the reference, while the 5% FA group achieved a 29.3% reduction. Although fly ash reduces Ca(OH)_2_ content, the resulting pore refinement and densification significantly limit CO_2_ ingress, enhancing carbonation resistance.

Notably, carbonation depth increased non-linearly with exposure time, especially in the reference specimens, where the rate accelerated significantly after 18 months. This behavior suggests that the coarse pore structure of the plain cement matrix facilitates deeper CO_2_ ingress over extended periods. In contrast, fly ash-modified mortars exhibited a more stable and moderate carbonation progression, highlighting their enhanced durability in CO_2_-rich environments.

It is worth noting that the substitution levels of 5–10% examined in the present study are considered more suitable for the Ptolemais fly ash, due to its high calcium content. Although higher replacement levels could further refine the pore structure, but would significantly reduce available portlandite (Ca(OH)_2_) and increase the formation of calcium-bearing phases more susceptible to carbonation. Thus, the low substitution levels balance pore refinement with the preservation of sufficient alkalinity, consistent with the observed carbonation behavior.”

### 3.7. Steel–Concrete Bond Behavior (Pull-Out Test)

**Figure 12:**
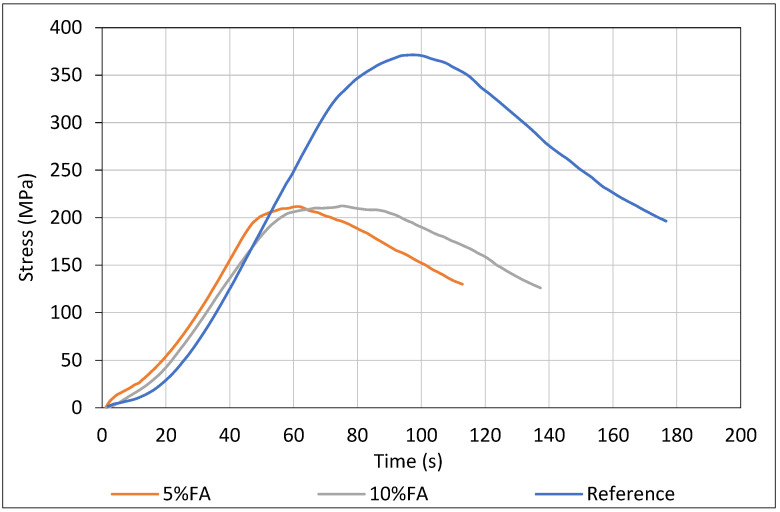
Steel–concrete bond after pull-out test on concrete specimens.

Bond strength tests (Figure 12) were conducted at 28 days, as this age typically represents the stage at which concrete attains its maximum early strength and bond capacity. Although bond strength can evolve with prolonged sulfate exposure, 28 days serves as a practical and widely accepted benchmark for assessing initial bond performance, particularly given the slower hydration kinetics of fly ash (FA) mixes.

The pull-out test results at 28 days revealed distinct differences in bond behavior among the three concrete mixes, highlighting the influence of fly ash content on early-age steel–concrete interaction. The reference mix, without fly ash, exhibited the highest peak bond stress (~370 MPa), considerably higher than the FA-containing mixes (~200–210 MPa), corresponding to a reduction of approximately 43–46%. This marked reduction in bond capacity is attributed to the lower early hydration product formation in FA mixes, as the pozzolanic activity of Ptolemais FA develops more slowly than that of OPC. Consequently, the matrix surrounding the embedded steel at 28 days is less mature and compact, limiting both mechanical interlock and chemical adhesion at the interface.

Among the FA mixes, the 10% FA group, despite having a similar peak strength to the 5% FA mix, displayed a broader post-peak plateau in the load–slip response, indicating more gradual bond degradation and higher energy dissipation. Nevertheless, both FA mixes reached peak bond strength at lower slip values compared to the reference, reflecting a shift toward a more friction-dependent and ductile failure mechanism.

Technically, incorporating fly ash at 5% and 10% levels compromises short-term bond performance, potentially necessitating extended curing or performance-enhancing additives to offset slower microstructural development. These findings underscore the importance of careful consideration of SCMs in bond-critical applications, especially when early-age performance is a design priority.

While this study provides valuable macroscopic insights through pull-out testing, it does not include microstructural characterization (e.g., SEM observations of the interfacial transition zone). Future studies should integrate mechanical testing with microscopic analysis to more conclusively elucidate the interfacial mechanisms governing steel–matrix bond performance.

It should be noted that pull-out tests were conducted only at 28 days. Since FA mixtures exhibit delayed strength development, long-term bond performance may differ from the early-age trends observed here. This represents a limitation of the present work, and future studies should investigate bond behavior at later curing ages (e.g., 90 days or beyond) to capture the full contribution of pozzolanic reactions.

This trend is attributed to the slow kinetics of the pozzolanic reaction, which consumes portlandite gradually, leading to a densified microstructure over time. The delayed reactivity is reflected in both the compressive and flexural strength values, as well as in the porosity parameters, where a reduction in total pore volume and refinement of the pore structure become more pronounced after 90 days of curing. These findings confirm that the inclusion of fly ash contributes to long-term enhancement of durability-related properties, albeit with a retarded initial performance due to the slower development of secondary C–S–H phases.

## 4. Discussion

This study examined the effects of incorporating 5% and 10% Ptolemais fly ash (FA) as partial cement replacement on the mechanical performance, durability, and corrosion behavior of mortar and concrete under standard and aggressive conditions. The observed trends are explained by the pozzolanic reaction, where calcium ions from the matrix react with the glassy FA phase, consuming portlandite and forming secondary C–S–H, which densifies the microstructure and refines pores, enhancing long-term durability.

Regarding the development of compressive and splitting tensile strengths the dual-phase behavior of FA is characterized by an initial period of strength suppression followed by notable long-term enhancement. Specifically, at early ages, FA-containing mixtures exhibited lower compressive and tensile strengths compared to the control mixture, particularly at higher FA contents. This early strength delay reflects the slower pozzolanic kinetics of calcium-rich FA compared to OPC, aligned with findings reported for similar high-calcium ashes in recent studies [47].

In the present study, the mixes exhibited reduced compressive and tensile strength, higher porosity, and increased reinforcement corrosion at early ages (up to 28 days), which can be attributed to the high water-to-cement ratios and the low substitution levels of calcium-rich Ptolemais fly ash. However, after 90 days, the presence of 5–10% Ca-rich fly ash significantly improved all these properties, including mechanical strength, pore refinement, and corrosion resistance. This delayed enhancement is attributed to ongoing pozzolanic reactions of the high-calcium fly ash, which densify the microstructure and mitigate durability-related issues. Although the high w/c ratio initially leads to lower early-age performance, the small addition of Ca-rich fly ash ensures a balance between workability, early-age performance, and long-term durability. These findings demonstrate that, even under challenging high w/c ratio, small amounts of Ca-rich FA can substantially enhance the performance of cementitious composites over time. Comparisons with calcium-rich fly ashes from similar geological sources indicate consistent behavior in long-term strength gain and durability improvements, further validating the results [48].

This behavior primarily reflects the early-stage dilution of reactive clinker by FA and the slower pozzolanic reactivity inherent to Ptolemais fly ash [49,50]. However, as curing advanced, this trend reversed, with FA mixtures surpassing the control in strength. This improvement reflects the activation of pozzolanic reactions in which amorphous in FA reacts with Ca(OH)_2_ to form additional C–S–H gel. The formation of secondary C–S–H contributes to matrix densification, pore refinement, and enhanced load-bearing capacity [51,52,53,54]. Moreover, the continued higher rate of strength gain in FA mixtures over extended curing periods underscores the sustained contribution of pozzolanic reactions to long-term performance. The parallel trends observed in splitting tensile strength indicate that both strength modalities benefit from progressive microstructural refinement.

In addition, Mercury Intrusion Porosimetry (MIP) findings corroborate the mechanical data by revealing significant changes in pore structure over time. Although FA mixtures initially exhibited higher total porosity due to slower early hydration, a pronounced refinement in pore structure developed with curing. Εspecially, the 10% FA mix evolved a finer and more discontinuous pore network, characterized by decreased capillary pore connectivity. This evolution directly corresponds to the in situ formation of additional C–S–H, which fills voids and disrupts pathways for fluid transport [55]. This pore refinement effect crucially impacts transport properties, reducing permeability and ion ingress, thereby enhancing durability [56].

**Figure 13:**
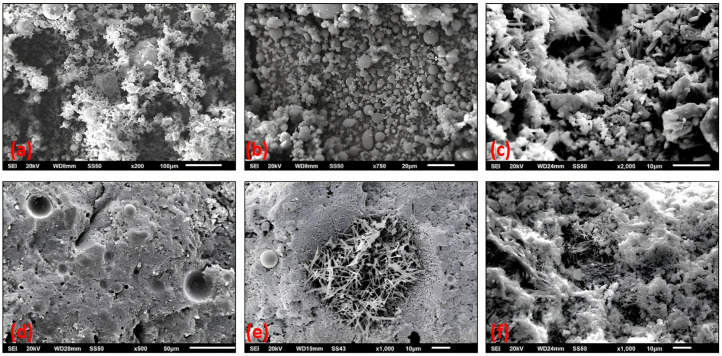
SEM micrographs showing raw fly ash with spherical cenospheres (**a**–**c**) and hardened fly ash mortars (**d**–**f**) featuring pore refinement, ettringite needles and CaCO_3_ deposits indicative of carbonation.

The SEM micrographs displayed in Figure 13 provide detailed insight into the microstructural characteristics of the raw fly ash and the hardened fly ash mortars. The top row images reveal the raw FA morphology, prominently featuring spherical cenospheres of varying sizes (middle image, ×750 magnification) surrounded by finer particles, which contribute to improved particle packing and initial porosity reduction. The left image (×200) shows the heterogeneous texture of raw FA aggregate, while the right image (×2000) highlights the intricate surface texture of FA, indicative of their pozzolanicity.

In the bottom row, the hardened FA mortars exhibit a significantly refined microstructure. At ×500 magnification (left), the matrix exhibits a dense, pore-reduced structure with isolated voids, demonstrating the effect of fly ash in filling and refining pore spaces. On the other hand, at ×1000 magnification image reveals abundant needle-like ettringite crystals embedded within the matrix, confirming ongoing sulfate hydration processes. Furthermore, the SEM images show densely packed hydration products with indicative morphologies of CaCO_3_ deposits, such as rhombohedral and granular formations within the matrix pores, evidencing the carbonation process.

These microstructural features collectively confirm two simultaneous phenomena: first, the pozzolanic and sulfate-related reactions promoted by the fly ash, leading to pore refinement and matrix densification; second, the formation of CaCO_3_ as part of carbonation, which further blocks pore connectivity and enhances resistance to ion ingress. This microstructural evolution underscores the mechanisms by which the fly ash contributes to improved durability against sulfate attack and carbonation-induced reinforcement corrosion. Importantly, while FA reduces portlandite content—typically lowering chemical buffering—its pore refinement effect dominates, limiting CO_2_ penetration and effectively slowing carbonation, a balance supported by recent mechanistic studies of calcium-rich fly ash systems [57].

Electrochemical monitoring of corrosion current density (*i*_corr_) revealed contrasting long-term behaviors between control and FA-containing specimens. The control samples initially exhibited relatively low corrosion activity, likely due to fast early densification; however, this protective effect waned over time as microstructural permeability allowed sulfate ions and moisture ingress. Conversely, FA-containing mixtures, especially those with the higher FA content, consistently maintained lower corrosion rates throughout exposure [58,59]. The enhanced corrosion resistance can be attributed to several interrelated mechanisms: refinement of the pore structure which impedes ion transport; the consumption of calcium hydroxide through pozzolanic reactions; and the formation of denser calcium silicate hydrate phases that effectively stabilize the cementitious matrix while promoting the passivation of embedded steel reinforcement [60,61,62].

Mass loss and corrosion rate measurements complemented the electrochemical data by demonstrating that FA-modified matrices endure less deterioration under aggressive exposures. While all specimens deteriorated over time, FA mixtures consistently exhibited slower damage progression, indicating that matrix densification and chemical stabilization imparted by FA are most effective during corrosion initiation and early propagation stages [63].

Interestingly, carbonation depth measurements revealed that the higher FA mix attained the lowest carbonation coefficient, outperforming the control despite its reduced portlandite content. This outcome can be explained by the dominating influence of permeability in governing carbonation resistance. Although the consumption of calcium hydroxide by FA reduces chemical buffering capacity, the denser pore structure limits CO_2_ ingress, effectively decelerating carbonation front penetration [64]. This finding highlights the necessity of assessing carbonation resistance through the combined effects of chemical and physical factors rather than solely Ca(OH)_2_ content. Although fly ash is typically associated with reduced alkalinity and increased susceptibility to carbonation, a 10% replacement with calcium-rich Ptolemais fly ash resulted in lower carbonation depth compared to plain cement mixes. This can be attributed to a combination of partial pore refinement at low substitution levels, the high CaO content of Ptolemais fly ash promoting additional calcium-bearing phases, and the higher water-to-cement ratios in the control mixes increasing their carbonation susceptibility. Together, these factors explain the enhanced carbonation resistance observed at early replacement levels.

Early-age pull-out tests showed a reduction in steel–mortar bond strength for FA mixtures compared to the control, due to an immature microstructure and lower hydration product volume at the interface [65,66]. However, the higher FA mix exhibited a broader post-peak softening plateau, indicating a more ductile bond failure, which may be advantageous under cyclic or seismic loading by enhancing energy dissipation [67,68].

In summary, 10% cement replacement with calcium-rich Ptolemais FA enhances long-term strength, microstructural densification, corrosion resistance and carbonation durability, despite slower early-age strength development. This makes it particularly suitable for structures in aggressive environments where durability is prioritized over early strength.

## 5. Conclusions

This study demonstrated that partial replacement of cement with calcium-rich Ptolemais fly ash (FA) markedly influences the long-term performance of cementitious composites under sulfate attack. While early-age properties were reduced, extended curing led to substantial improvements in mechanical performance, microstructural refinement, and durability. The main conclusions can be summarized as follows:

- Strength development: The 10% FA mixture was weaker than the control at 28 days but surpassed it by more than 20% at 180 days.
- Porosity reduction: Mercury intrusion porosimetry showed a 30–35% decrease in total porosity between 28 and 180 days in FA composites.
- Durability gains: After 12 months of sulfate exposure, corrosion rates in FA mixtures were up to 10–11 times lower than in the control. Furthermore, Ptolemais FA groups exhibited the lowest carbonation coefficient, highlighting the dominant role of permeability over chemical buffering.
- Bond strength: Early-age bond strength declined by ~45%, while the increased post-peak ductility may benefit energy-dissipating structures.

Overall, calcium-rich Ptolemais fly ash at 10% replacement is an effective SCM that, despite short-term drawbacks, provides significant long-term enhancements in strength, permeability, and corrosion resistance, making it suitable for sustainable and durable structural applications.

## Figures and Tables

**Table 1:** Chemical composition of fly ash (FA) and Ordinary Portland cement (OPC) used for specimen preparation (% wt. by cement mass).

Oxides	FA (%)	CEM I 42.5 N (%)
SiO_2_	34.73	18.89
Al_2_O_3_	15.78	4.65
Fe_2_O_3_	6.20	3.49
CaO	27.41	61.55
MgO	3.04	3.30
K_2_O	1.17	0.67
Na_2_O	0.46	0.21
SO_3_	3.19	2.52
CaO_free_	11.81	0.97
LOI	1.89	3.39
I.R	26.42	0.19
Glassy Phase	25.26	—

Note: ‘—’ indicates data not applicable.

**Table 2:** Material quantities (kg/m^3^) and weight ratios in concrete mixtures.

Material	Reference	5% wt. FA	10% wt. FA
Cement	376	357	338
Fly ash (FA)	—	18.8	37.6
Fine Aggregate (0–4 mm)	715	715	715
Coarse Aggregate (4–10 mm)	611	611	611
Coarse Aggregate (8–20 mm)	433	433	433
Water	244	256	271
Water: (Cement + FA)	0.65	0.68	0.72

## Data Availability

The original contributions presented in this study are included in the article material. Further inquiries can be directed to the corresponding author.
